# Diethyl 2-[(5-meth­oxy-2-methyl-1-phenyl­sulfonyl-1*H*-indol-3-yl)methyl­ene]malonate

**DOI:** 10.1107/S1600536809019369

**Published:** 2009-05-29

**Authors:** T. Kavitha, M. Thenmozhi, V. Dhayalan, A. K. Mohanakrishnan, M. N. Ponnuswamy

**Affiliations:** a Centre of Advanced Study in Crystallography and Biophysics, University of Madras, Guindy Campus, Chennai 600 025, India; b Department of Organic Chemistry, University of Madras, Guindy Campus, Chennai 600 025, India

## Abstract

In the title compound, C_24_H_25_NO_7_S, the sulfonyl-bound phenyl ring is approximately perpendicular to the indole ring system [dihedral angle = 87.72 (5)°]. The methyl group of one of the ester units is disordered over two positions with occupancies of 0.527 (13) and 0.473 (13). An intra­molecular C—H⋯O hydrogen bond is observed. In the crystal structure, mol­ecules are linked into a ribbon structure running along the *c* axis by inter­molecular C—H⋯O hydrogen bonds and C—H⋯π inter­actions involving the pyrrole ring.

## Related literature

For general background on indoles, see: El-Sayed *et al.* (1986 ▶); Farhanullah *et al.* (2004 ▶); Okabe & Adachi (1998 ▶); Schollmeyer *et al.* (1995 ▶). For the Thorpe–Ingold effect, see: Bassindale (1984 ▶). For hybridization, see: Beddoes *et al.* (1986 ▶). For a related structure, see: Chakkaravarthi *et al.* (2008 ▶).
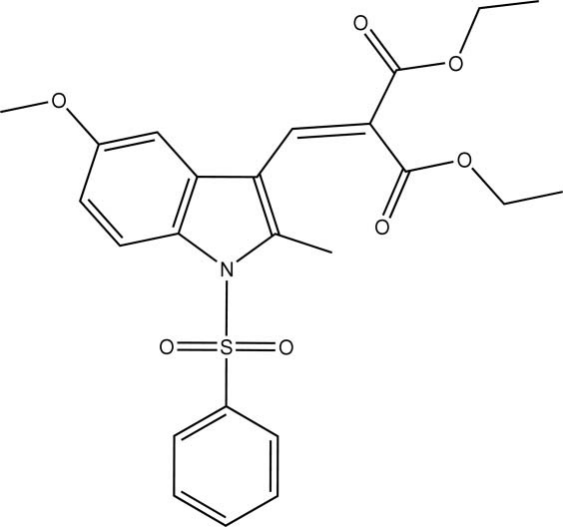


## Experimental

### 

#### Crystal data

C_24_H_25_NO_7_S
*M* *_r_* = 471.51
Triclinic, 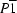

*a* = 8.7597 (2) Å
*b* = 10.9029 (2) Å
*c* = 12.5186 (3) Å
α = 88.402 (1)°
β = 79.723 (1)°
γ = 83.635 (2)°
*V* = 1169.13 (4) Å^3^
*Z* = 2
Mo *K*α radiation
μ = 0.18 mm^−1^
*T* = 293 K
0.22 × 0.18 × 0.15 mm

#### Data collection

Bruker Kappa APEXII area-detector diffractometer
Absorption correction: multi-scan (*SADABS*; Sheldrick, 2001 ▶) *T*_min_ = 0.964, *T*_max_ = 0.971
27454 measured reflections
6218 independent reflections
4628 reflections with *I* > 2σ(*I*)
*R*_int_ = 0.023

#### Refinement

*R*[*F* ^2^ > 2σ(*F* ^2^)] = 0.053
*wR*(*F* ^2^) = 0.176
*S* = 1.07
6218 reflections
309 parameters
16 restraints
H-atom parameters constrained
Δρ_max_ = 0.66 e Å^−3^
Δρ_min_ = −0.45 e Å^−3^

### 

Data collection: *APEX2* (Bruker, 2004 ▶); cell refinement: *SAINT* (Bruker, 2004 ▶); data reduction: *SAINT*; program(s) used to solve structure: *SHELXS97* (Sheldrick, 2008 ▶); program(s) used to refine structure: *SHELXL97* (Sheldrick, 2008 ▶); molecular graphics: *ORTEP-3* (Farrugia, 1997 ▶); software used to prepare material for publication: *SHELXL97* and *PLATON* (Spek, 2009 ▶).

## Supplementary Material

Crystal structure: contains datablocks I, global. DOI: 10.1107/S1600536809019369/ci2774sup1.cif

Structure factors: contains datablocks I. DOI: 10.1107/S1600536809019369/ci2774Isup2.hkl

Additional supplementary materials:  crystallographic information; 3D view; checkCIF report

## Figures and Tables

**Table 1:** Hydrogen-bond geometry (Å, °) *Cg*1 is the centroid of the N1/C2–C5 ring.

*D*—H⋯*A*	*D*—H	H⋯*A*	*D*⋯*A*	*D*—H⋯*A*
C6—H6⋯O1	0.93	2.32	2.901 (3)	120
C14—H14⋯O5^i^	0.93	2.42	3.349 (3)	173
C25—H25*C*⋯*Cg*1^ii^	0.96	2.92	3.515 (3)	140

Symmetry codes: (i) 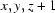; (ii) 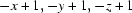.

**Fig. 1.**
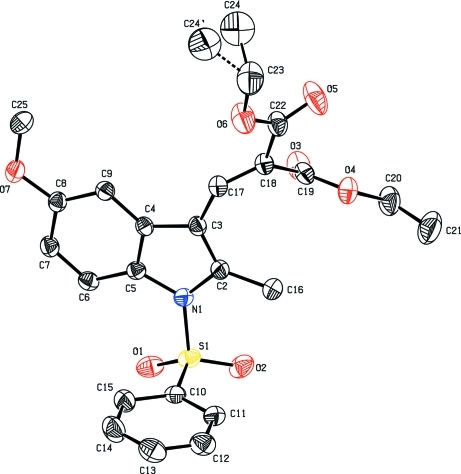
Molecular structure of the title compound. Displacement ellipsoids are drawn at the 20% probability level. H atoms have been omitted for clarity. Both disorder components are shown.

**Fig. 2.**
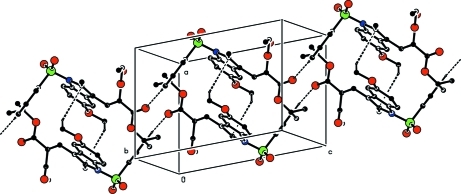
Crystal packing of the title compound. H atoms not involved in hydrogen bonding (dashed lines) have been omitted.

## supplementary crystallographic information

### Comment

Indoles form an integral part of many natural products and possess potentially reactive sites to perform variety of chemical reactions to generate molecular diversity (Farhanullah *et al.*, 2004). Many of the indole derivatives are found to possess antibacterial (Okabe & Adachi, 1998), antitumour (Schollmeyer *et al.*, 1995) and antimicrobial (El-Sayed *et al.*, 1986) activities.

The bond angles around atom S1 show significant deviation from regular tetrahedral geometry. The widening of O1—S1—O2 [120.42 (11)°] and the narrowing of the angle N1—S1—C10 [104.43 (9)°] are attributed to the Thorpe-Ingold effect (Bassindale, 1984). The indole ring system is planar. The bond lengths and angles are comparable to those observed in a related structure (Chakkaravarthi *et al.*, 2008). The sum of bond angles around atom N1 (357.9°) indicates that the atom N1 is in *sp*^2^ hybridized state (Beddoes *et al.*, 1986). The sulfonyl bound phenyl ring (C10–C15) is approximately perpendicular to the indole ring system, with the dihedral angle being 87.72 (5)°. The C18–C21/O3/O4 ester group shows an extended conformation, as is evident from the torsion angles C18—C19—O4—C20 = 179.8 (2)° and C19—O4—C20—C21 = -169.9 (3)°. The terminal methyl group of the other ester group is disordered over two positions. An intramolecular C6—H6···O1 hydrogen bond is observed.

In the crystal structure, the molecules are linked into a zigzag C(12) chain running along the *c* axis by intermolecular C—H···O hydrogen bonds involving atoms C14 and O5 (Table 1). The inversion-related molecules of adjacent chains are linked via weak C—H···π interactions involving the N1/C2–C5 ring (centroid *Cg*1) to form a ribbon structure (Fig. 2).

### Experimental

A solution of TiCl_4_ (0.37 ml, 3.34 mmol) in dry dichloromethane (DCM; 20 ml) was added drop by drop into dry DCM (10 ml) kept at 273 K and stirred at the same temperature for 45 min. To this, a solution of 5-methoxy-2-methyl-1-(phenylsulfonyl)-1*H*-indole-3-carbaldehyde (0.5 g, 1.52 mmol) and diethylmalonate (0.24 ml, 1.60 mmol) in dry DCM (20 ml) was slowly added. After the addition was completed, the reaction mixture was stirred at 273 K for 1 h. A solution of pyridine (0.6 ml, 6.08 mmol) in dry DCM (20 ml) was then added dropwise for 30 min. The content was stirred at 273 K for 12 h and then at room temperature for 48 h. The reaction mass was quenched with water and extracted with CHCl_3_ (2 × 10 ml). The organic layer was washed with 0.5 *M* HCl (2 × 20 ml) followed by brine solution (2 × 20 ml). Removal of solvent followed by flash column chromatographic purification (n-hexane–ethyl acetate 97:3) afforded the title compound as a yellow solid. Single crystals were obtained by recrystallizing the compound from n-hexane–ethyl acetate (97:3).

### Refinement

The methyl group at the end of one of ethyl carboxylate chains is disordered over two positions (C24 and C24') with refined occupancies of 0.527 (13) and 0.473 (13). The C23—C24 and C23—C24'bond distances were restrained to be 1.50 (5) Å. The components of the anisotropic displacement parameters of atoms C13, C14, C23 and O6 in the direction of the bond between them were restrained to be equal within an effective standard deviation of 0.001. The *U^ij^* parameters of atoms C24 and C24' were restrained to an approximate isotropic behaviour. H atoms were positioned geometrically and refined using riding model with C-H = 0.93-0.97 Å and *U*_iso_(H) = 1.2*U*_eq_(C) and 1.5*U*_eq_(C_methyl_).

### Crystal data

**Table:**

C_24_H_25_NO_7_S	*Z* = 2
*M**_r_* = 471.51	*F*(000) = 496
Triclinic, *P*1	*D*_x_ = 1.339 Mg m^−^^3^
Hall symbol: -P 1	Mo *K*α radiation, λ = 0.71073 Å
*a* = 8.7597 (2) Å	Cell parameters from 6218 reflections
*b* = 10.9029 (2) Å	θ = 1.6–29.1°
*c* = 12.5186 (3) Å	µ = 0.18 mm^−^^1^
α = 88.402 (1)°	*T* = 293 K
β = 79.723 (1)°	Block, yellow
γ = 83.635 (2)°	0.22 × 0.18 × 0.15 mm
*V* = 1169.13 (4) Å^3^	

### Data collection

**Table:**

Bruker Kappa-APEXII area-detector diffractometer	6218 independent reflections
Radiation source: fine-focus sealed tube	4628 reflections with *I* > 2σ(*I*)
graphite	*R*_int_ = 0.023
ω and φ scans	θ_max_ = 29.1°, θ_min_ = 1.7°
Absorption correction: multi-scan (*SADABS*; Sheldrick, 2001)	*h* = −11→11
*T*_min_ = 0.964, *T*_max_ = 0.971	*k* = −14→14
27454 measured reflections	*l* = −17→17

### Refinement

**Table:**

Refinement on *F*^2^	Primary atom site location: structure-invariant direct methods
Least-squares matrix: full	Secondary atom site location: difference Fourier map
*R*[*F*^2^ > 2σ(*F*^2^)] = 0.053	Hydrogen site location: inferred from neighbouring sites
*wR*(*F*^2^) = 0.176	H-atom parameters constrained
*S* = 1.07	*w* = 1/[σ^2^(*F*_o_^2^) + (0.09*P*)^2^ + 0.3748*P*] where *P* = (*F*_o_^2^ + 2*F*_c_^2^)/3
6218 reflections	(Δ/σ)_max_ = 0.001
309 parameters	Δρ_max_ = 0.66 e Å^−^^3^
16 restraints	Δρ_min_ = −0.45 e Å^−^^3^

### Special details

**Table:**

Geometry. All e.s.d.'s (except the e.s.d. in the dihedral angle between two l.s. planes) are estimated using the full covariance matrix. The cell e.s.d.'s are taken into account individually in the estimation of e.s.d.'s in distances, angles and torsion angles; correlations between e.s.d.'s in cell parameters are only used when they are defined by crystal symmetry. An approximate (isotropic) treatment of cell e.s.d.'s is used for estimating e.s.d.'s involving l.s. planes.
Refinement. Refinement of *F*^2^ against ALL reflections. The weighted *R*-factor *wR* and goodness of fit *S* are based on *F*^2^, conventional *R*-factors *R* are based on *F*, with *F* set to zero for negative *F*^2^. The threshold expression of *F*^2^ > σ(*F*^2^) is used only for calculating *R*-factors(gt) *etc*. and is not relevant to the choice of reflections for refinement. *R*-factors based on *F*^2^ are statistically about twice as large as those based on *F*, and *R*- factors based on ALL data will be even larger.

### Fractional atomic coordinates and isotropic or equivalent isotropic displacement parameters (Å^2^)

**Table:**

	*x*	*y*	*z*	*U*_iso_*/*U*_eq_	Occ. (<1)
C2	0.1929 (2)	0.26604 (18)	0.44185 (16)	0.0459 (4)	
C3	0.2837 (2)	0.35252 (17)	0.39343 (15)	0.0423 (4)	
C4	0.2631 (2)	0.45572 (17)	0.46608 (14)	0.0390 (4)	
C5	0.1556 (2)	0.43026 (17)	0.55745 (15)	0.0399 (4)	
C6	0.1105 (2)	0.51428 (18)	0.64167 (15)	0.0462 (4)	
H6	0.0372	0.4979	0.7023	0.055*	
C7	0.1787 (2)	0.62274 (19)	0.63165 (16)	0.0482 (4)	
H7	0.1523	0.6797	0.6876	0.058*	
C8	0.2861 (2)	0.64990 (18)	0.54028 (16)	0.0442 (4)	
C9	0.3283 (2)	0.56763 (18)	0.45571 (15)	0.0427 (4)	
H9	0.3979	0.5861	0.3937	0.051*	
C10	0.1592 (2)	0.15262 (18)	0.70459 (16)	0.0461 (4)	
C11	0.2091 (3)	0.0301 (2)	0.68271 (18)	0.0550 (5)	
H11	0.1655	−0.0122	0.6342	0.066*	
C12	0.3239 (3)	−0.0290 (2)	0.7332 (2)	0.0682 (6)	
H12	0.3584	−0.1117	0.7190	0.082*	
C13	0.3875 (3)	0.0341 (3)	0.8045 (2)	0.0745 (7)	
H13	0.4654	−0.0061	0.8384	0.089*	
C14	0.3377 (4)	0.1551 (3)	0.8262 (2)	0.0756 (7)	
H14	0.3818	0.1968	0.8749	0.091*	
C15	0.2218 (3)	0.2167 (2)	0.77638 (19)	0.0611 (6)	
H15	0.1873	0.2993	0.7911	0.073*	
C16	0.1853 (4)	0.1390 (2)	0.4038 (2)	0.0672 (6)	
H16A	0.0883	0.1354	0.3790	0.101*	
H16B	0.1924	0.0813	0.4625	0.101*	
H16C	0.2704	0.1183	0.3452	0.101*	
C17	0.3945 (2)	0.34685 (18)	0.29260 (16)	0.0465 (4)	
H17	0.4866	0.3808	0.2952	0.056*	
C18	0.3860 (2)	0.30077 (18)	0.19641 (16)	0.0470 (4)	
C19	0.2432 (2)	0.2538 (2)	0.17047 (16)	0.0506 (5)	
C20	0.1390 (3)	0.0829 (3)	0.1115 (3)	0.0788 (8)	
H20A	0.1112	0.1196	0.0452	0.095*	
H20B	0.0494	0.0975	0.1693	0.095*	
C21	0.1827 (5)	−0.0488 (3)	0.0973 (3)	0.1081 (12)	
H21A	0.0969	−0.0865	0.0789	0.162*	
H21B	0.2087	−0.0846	0.1636	0.162*	
H21C	0.2714	−0.0625	0.0400	0.162*	
C22	0.5148 (3)	0.3041 (2)	0.10308 (19)	0.0587 (5)	
C23	0.7754 (4)	0.3516 (4)	0.0435 (3)	0.1053 (13)	
H23A	0.7917	0.2818	−0.0051	0.126*	
H23B	0.8686	0.3537	0.0748	0.126*	
C24	0.7462 (13)	0.4658 (8)	−0.0169 (9)	0.136 (4)	0.527 (13)
H24A	0.8345	0.4753	−0.0730	0.204*	0.527 (13)
H24B	0.7299	0.5346	0.0315	0.204*	0.527 (13)
H24C	0.6551	0.4626	−0.0491	0.204*	0.527 (13)
C24'	0.8132 (13)	0.4810 (6)	0.0454 (10)	0.125 (4)	0.473 (13)
H24D	0.9014	0.4925	−0.0102	0.187*	0.473 (13)
H24E	0.8375	0.4977	0.1150	0.187*	0.473 (13)
H24F	0.7251	0.5364	0.0326	0.187*	0.473 (13)
C25	0.4367 (3)	0.8015 (2)	0.4473 (2)	0.0655 (6)	
H25A	0.4678	0.8809	0.4593	0.098*	
H25B	0.3789	0.8073	0.3887	0.098*	
H25C	0.5277	0.7433	0.4291	0.098*	
N1	0.10926 (19)	0.31258 (15)	0.54194 (13)	0.0450 (4)	
O1	−0.07993 (18)	0.31630 (15)	0.71397 (14)	0.0640 (4)	
O2	−0.05720 (19)	0.14123 (16)	0.59054 (15)	0.0668 (5)	
O3	0.11914 (19)	0.31388 (18)	0.17716 (15)	0.0703 (5)	
O4	0.27014 (18)	0.13750 (15)	0.13834 (14)	0.0620 (4)	
O5	0.5020 (3)	0.2806 (3)	0.01285 (16)	0.1019 (8)	
O6	0.6431 (2)	0.3383 (2)	0.12855 (16)	0.0883 (7)	
O7	0.34224 (19)	0.76187 (14)	0.54229 (13)	0.0588 (4)	
S1	0.01286 (6)	0.23002 (5)	0.64069 (4)	0.04951 (16)	

### Atomic displacement parameters (Å^2^)

**Table:**

	*U*^11^	*U*^22^	*U*^33^	*U*^12^	*U*^13^	*U*^23^
C2	0.0544 (10)	0.0432 (10)	0.0434 (9)	−0.0068 (8)	−0.0163 (8)	0.0002 (8)
C3	0.0455 (9)	0.0434 (9)	0.0398 (9)	−0.0034 (7)	−0.0130 (7)	−0.0031 (7)
C4	0.0372 (8)	0.0437 (9)	0.0376 (8)	−0.0026 (7)	−0.0115 (7)	−0.0017 (7)
C5	0.0388 (8)	0.0407 (9)	0.0412 (9)	−0.0036 (7)	−0.0111 (7)	0.0038 (7)
C6	0.0467 (10)	0.0499 (10)	0.0397 (9)	−0.0027 (8)	−0.0031 (7)	0.0012 (8)
C7	0.0522 (10)	0.0478 (10)	0.0436 (10)	−0.0023 (8)	−0.0064 (8)	−0.0076 (8)
C8	0.0439 (9)	0.0434 (10)	0.0473 (10)	−0.0062 (7)	−0.0128 (8)	−0.0022 (8)
C9	0.0397 (8)	0.0478 (10)	0.0414 (9)	−0.0075 (7)	−0.0079 (7)	−0.0001 (7)
C10	0.0471 (9)	0.0492 (10)	0.0428 (9)	−0.0094 (8)	−0.0088 (8)	0.0095 (8)
C11	0.0635 (12)	0.0507 (11)	0.0528 (11)	−0.0101 (9)	−0.0147 (10)	0.0068 (9)
C12	0.0740 (15)	0.0624 (14)	0.0659 (15)	0.0076 (12)	−0.0171 (12)	0.0075 (12)
C13	0.0711 (15)	0.0868 (14)	0.0675 (15)	0.0054 (13)	−0.0279 (13)	0.0119 (13)
C14	0.0849 (18)	0.0880 (15)	0.0622 (14)	−0.0091 (14)	−0.0347 (14)	−0.0049 (13)
C15	0.0748 (15)	0.0584 (13)	0.0537 (12)	−0.0061 (11)	−0.0216 (11)	−0.0009 (10)
C16	0.1018 (19)	0.0461 (11)	0.0566 (13)	−0.0175 (12)	−0.0155 (13)	−0.0015 (10)
C17	0.0470 (10)	0.0458 (10)	0.0471 (10)	−0.0044 (8)	−0.0089 (8)	−0.0051 (8)
C18	0.0461 (10)	0.0489 (10)	0.0460 (10)	−0.0036 (8)	−0.0078 (8)	−0.0071 (8)
C19	0.0474 (10)	0.0630 (12)	0.0409 (9)	−0.0042 (9)	−0.0062 (8)	−0.0079 (9)
C20	0.0690 (16)	0.093 (2)	0.0817 (18)	−0.0299 (14)	−0.0183 (13)	−0.0193 (15)
C21	0.112 (3)	0.092 (2)	0.126 (3)	−0.050 (2)	−0.008 (2)	−0.026 (2)
C22	0.0522 (11)	0.0685 (14)	0.0543 (12)	−0.0114 (10)	−0.0009 (9)	−0.0172 (10)
C23	0.0772 (19)	0.128 (3)	0.105 (2)	−0.044 (2)	0.0246 (15)	−0.035 (2)
C24	0.135 (4)	0.136 (4)	0.136 (4)	−0.0144 (11)	−0.0234 (12)	0.0001 (10)
C24'	0.125 (4)	0.125 (4)	0.125 (4)	−0.0152 (11)	−0.0213 (12)	0.0003 (10)
C25	0.0642 (14)	0.0622 (14)	0.0728 (15)	−0.0255 (11)	−0.0089 (11)	0.0043 (11)
N1	0.0512 (9)	0.0430 (8)	0.0429 (8)	−0.0096 (7)	−0.0122 (7)	0.0052 (6)
O1	0.0498 (8)	0.0646 (10)	0.0700 (10)	−0.0029 (7)	0.0056 (7)	0.0129 (8)
O2	0.0590 (9)	0.0645 (10)	0.0867 (12)	−0.0265 (8)	−0.0296 (8)	0.0149 (9)
O3	0.0480 (8)	0.0871 (12)	0.0744 (11)	0.0051 (8)	−0.0117 (8)	−0.0208 (9)
O4	0.0545 (8)	0.0616 (9)	0.0736 (10)	−0.0097 (7)	−0.0169 (7)	−0.0169 (8)
O5	0.0799 (13)	0.169 (2)	0.0583 (11)	−0.0429 (14)	0.0093 (9)	−0.0447 (13)
O6	0.0620 (11)	0.1307 (18)	0.0742 (12)	−0.0402 (11)	0.0049 (8)	−0.0285 (12)
O7	0.0675 (9)	0.0513 (8)	0.0599 (9)	−0.0203 (7)	−0.0080 (7)	−0.0071 (7)
S1	0.0422 (3)	0.0513 (3)	0.0567 (3)	−0.0116 (2)	−0.0114 (2)	0.0123 (2)

### Geometric parameters (Å, °)

**Table:**

C2—C3	1.360 (3)	C17—H17	0.93
C2—N1	1.411 (3)	C18—C22	1.475 (3)
C2—C16	1.490 (3)	C18—C19	1.493 (3)
C3—C4	1.442 (2)	C19—O3	1.196 (3)
C3—C17	1.447 (3)	C19—O4	1.324 (3)
C4—C5	1.388 (3)	C20—O4	1.445 (3)
C4—C9	1.396 (3)	C20—C21	1.452 (5)
C5—C6	1.390 (3)	C20—H20A	0.97
C5—N1	1.416 (2)	C20—H20B	0.97
C6—C7	1.376 (3)	C21—H21A	0.96
C6—H6	0.93	C21—H21B	0.96
C7—C8	1.393 (3)	C21—H21C	0.96
C7—H7	0.93	C22—O5	1.193 (3)
C8—O7	1.368 (2)	C22—O6	1.316 (3)
C8—C9	1.379 (3)	C23—O6	1.445 (3)
C9—H9	0.93	C23—C24	1.461 (5)
C10—C15	1.376 (3)	C23—C24'	1.486 (5)
C10—C11	1.378 (3)	C23—H23A	0.97
C10—S1	1.756 (2)	C23—H23B	0.97
C11—C12	1.373 (3)	C24—H24A	0.96
C11—H11	0.93	C24—H24B	0.96
C12—C13	1.370 (4)	C24—H24C	0.96
C12—H12	0.93	C24'—H24D	0.96
C13—C14	1.362 (4)	C24'—H24E	0.96
C13—H13	0.93	C24'—H24F	0.96
C14—C15	1.386 (3)	C25—O7	1.409 (3)
C14—H14	0.93	C25—H25A	0.96
C15—H15	0.93	C25—H25B	0.96
C16—H16A	0.96	C25—H25C	0.96
C16—H16B	0.96	N1—S1	1.6690 (16)
C16—H16C	0.96	O1—S1	1.4141 (17)
C17—C18	1.335 (3)	O2—S1	1.4181 (17)
			
C3—C2—N1	108.67 (16)	O4—C19—C18	111.98 (17)
C3—C2—C16	128.0 (2)	O4—C20—C21	108.4 (3)
N1—C2—C16	123.20 (19)	O4—C20—H20A	110.0
C2—C3—C4	107.82 (17)	C21—C20—H20A	110.0
C2—C3—C17	129.75 (18)	O4—C20—H20B	110.0
C4—C3—C17	122.24 (17)	C21—C20—H20B	110.0
C5—C4—C9	120.75 (17)	H20A—C20—H20B	108.4
C5—C4—C3	108.18 (16)	C20—C21—H21A	109.5
C9—C4—C3	131.05 (17)	C20—C21—H21B	109.5
C4—C5—C6	121.34 (17)	H21A—C21—H21B	109.5
C4—C5—N1	106.95 (16)	C20—C21—H21C	109.5
C6—C5—N1	131.70 (17)	H21A—C21—H21C	109.5
C7—C6—C5	117.28 (18)	H21B—C21—H21C	109.5
C7—C6—H6	121.4	O5—C22—O6	123.5 (2)
C5—C6—H6	121.4	O5—C22—C18	122.8 (2)
C6—C7—C8	122.06 (18)	O6—C22—C18	113.66 (19)
C6—C7—H7	119.0	O6—C23—C24	109.3 (5)
C8—C7—H7	119.0	O6—C23—C24'	107.6 (5)
O7—C8—C9	124.44 (18)	O6—C23—H23A	109.8
O7—C8—C7	114.95 (17)	C24—C23—H23A	109.8
C9—C8—C7	120.60 (18)	C24'—C23—H23A	140.2
C8—C9—C4	117.94 (17)	O6—C23—H23B	109.8
C8—C9—H9	121.0	C24—C23—H23B	109.8
C4—C9—H9	121.0	C24'—C23—H23B	70.5
C15—C10—C11	121.2 (2)	H23A—C23—H23B	108.3
C15—C10—S1	118.66 (17)	C23—C24—H24A	109.5
C11—C10—S1	120.13 (16)	C23—C24—H24B	109.5
C12—C11—C10	119.4 (2)	H24A—C24—H24B	109.5
C12—C11—H11	120.3	C23—C24—H24C	109.5
C10—C11—H11	120.3	H24A—C24—H24C	109.5
C13—C12—C11	119.9 (2)	H24B—C24—H24C	109.5
C13—C12—H12	120.1	C23—C24'—H24D	109.5
C11—C12—H12	120.1	C23—C24'—H24E	109.5
C14—C13—C12	120.7 (2)	H24D—C24'—H24E	109.5
C14—C13—H13	119.7	C23—C24'—H24F	109.5
C12—C13—H13	119.7	H24D—C24'—H24F	109.5
C13—C14—C15	120.5 (2)	H24E—C24'—H24F	109.5
C13—C14—H14	119.7	O7—C25—H25A	109.5
C15—C14—H14	119.7	O7—C25—H25B	109.5
C10—C15—C14	118.3 (2)	H25A—C25—H25B	109.5
C10—C15—H15	120.8	O7—C25—H25C	109.5
C14—C15—H15	120.8	H25A—C25—H25C	109.5
C2—C16—H16A	109.5	H25B—C25—H25C	109.5
C2—C16—H16B	109.5	C2—N1—C5	108.32 (15)
H16A—C16—H16B	109.5	C2—N1—S1	125.65 (13)
C2—C16—H16C	109.5	C5—N1—S1	123.91 (13)
H16A—C16—H16C	109.5	C19—O4—C20	116.42 (19)
H16B—C16—H16C	109.5	C22—O6—C23	119.1 (2)
C18—C17—C3	130.42 (19)	C8—O7—C25	117.79 (17)
C18—C17—H17	114.8	O1—S1—O2	120.42 (11)
C3—C17—H17	114.8	O1—S1—N1	106.20 (9)
C17—C18—C22	121.49 (19)	O2—S1—N1	107.38 (10)
C17—C18—C19	123.87 (18)	O1—S1—C10	108.77 (10)
C22—C18—C19	114.37 (17)	O2—S1—C10	108.50 (10)
O3—C19—O4	123.8 (2)	N1—S1—C10	104.43 (9)
O3—C19—C18	124.2 (2)		
			
N1—C2—C3—C4	2.4 (2)	C17—C18—C19—O4	−122.7 (2)
C16—C2—C3—C4	−173.8 (2)	C22—C18—C19—O4	63.2 (2)
N1—C2—C3—C17	177.27 (18)	C17—C18—C22—O5	−168.7 (3)
C16—C2—C3—C17	1.0 (3)	C19—C18—C22—O5	5.6 (4)
C2—C3—C4—C5	−1.4 (2)	C17—C18—C22—O6	9.7 (3)
C17—C3—C4—C5	−176.76 (16)	C19—C18—C22—O6	−176.0 (2)
C2—C3—C4—C9	−179.75 (18)	C3—C2—N1—C5	−2.5 (2)
C17—C3—C4—C9	4.9 (3)	C16—C2—N1—C5	173.94 (19)
C9—C4—C5—C6	−0.6 (3)	C3—C2—N1—S1	−166.42 (13)
C3—C4—C5—C6	−179.14 (16)	C16—C2—N1—S1	10.0 (3)
C9—C4—C5—N1	178.41 (15)	C4—C5—N1—C2	1.58 (19)
C3—C4—C5—N1	−0.13 (19)	C6—C5—N1—C2	−179.56 (19)
C4—C5—C6—C7	−1.1 (3)	C4—C5—N1—S1	165.83 (13)
N1—C5—C6—C7	−179.83 (18)	C6—C5—N1—S1	−15.3 (3)
C5—C6—C7—C8	1.4 (3)	O3—C19—O4—C20	−0.6 (3)
C6—C7—C8—O7	179.45 (18)	C18—C19—O4—C20	179.8 (2)
C6—C7—C8—C9	0.0 (3)	C21—C20—O4—C19	−169.9 (3)
O7—C8—C9—C4	178.90 (17)	O5—C22—O6—C23	1.6 (5)
C7—C8—C9—C4	−1.7 (3)	C18—C22—O6—C23	−176.8 (3)
C5—C4—C9—C8	2.0 (3)	C24—C23—O6—C22	75.7 (6)
C3—C4—C9—C8	−179.87 (18)	C24'—C23—O6—C22	121.2 (6)
C15—C10—C11—C12	−0.2 (3)	C9—C8—O7—C25	6.9 (3)
S1—C10—C11—C12	179.85 (18)	C7—C8—O7—C25	−172.56 (19)
C10—C11—C12—C13	0.0 (4)	C2—N1—S1—O1	−167.59 (16)
C11—C12—C13—C14	0.2 (4)	C5—N1—S1—O1	30.90 (18)
C12—C13—C14—C15	−0.1 (5)	C2—N1—S1—O2	−37.54 (18)
C11—C10—C15—C14	0.3 (4)	C5—N1—S1—O2	160.94 (15)
S1—C10—C15—C14	−179.8 (2)	C2—N1—S1—C10	77.54 (17)
C13—C14—C15—C10	−0.1 (4)	C5—N1—S1—C10	−83.98 (16)
C2—C3—C17—C18	41.7 (3)	C15—C10—S1—O1	−34.6 (2)
C4—C3—C17—C18	−144.1 (2)	C11—C10—S1—O1	145.35 (17)
C3—C17—C18—C22	−179.7 (2)	C15—C10—S1—O2	−167.21 (18)
C3—C17—C18—C19	6.6 (4)	C11—C10—S1—O2	12.7 (2)
C17—C18—C19—O3	57.7 (3)	C15—C10—S1—N1	78.50 (19)
C22—C18—C19—O3	−116.4 (2)	C11—C10—S1—N1	−101.59 (18)

### Hydrogen-bond geometry (Å, °)

**Table:**

*D*—H···*A*	*D*—H	H···*A*	*D*···*A*	*D*—H···*A*
C6—H6···O1	0.93	2.32	2.901 (3)	120
C14—H14···O5^i^	0.93	2.42	3.349 (3)	173
C25—H25C···Cg1^ii^	0.96	2.92	3.515 (3)	140

Symmetry codes:  (i) *x*, *y*, *z*+1; (ii) −*x*+1, −*y*+1, −*z*+1.
